# Evaluation of SOFA-based models for predicting mortality in the ICU: A systematic review

**DOI:** 10.1186/cc7160

**Published:** 2008-12-17

**Authors:** Lilian Minne, Ameen Abu-Hanna, Evert de Jonge

**Affiliations:** 1 Department of Medical Informatics, Academic Medical Center, Meibergdreef 9, 1105 AZ, Amsterdam, The Netherlands; 2 Intensive Care Department, Academic Medical Center, Meibergdreef 9, 1105 AZ, Amsterdam, The Netherlands

## Abstract

**Introduction:**

To systematically review studies evaluating the performance of Sequential Organ Failure Assessment (SOFA)-based models for predicting mortality in patients in the intensive care unit (ICU).

**Methods:**

Medline, EMBASE and other databases were searched for English-language articles with the major objective of evaluating the prognostic performance of SOFA-based models in predicting mortality in surgical and/or medical ICU admissions. The quality of each study was assessed based on a quality framework for prognostic models.

**Results:**

Eighteen articles met all inclusion criteria. The studies differed widely in the SOFA derivatives used and in their methods of evaluation. Ten studies reported about developing a probabilistic prognostic model, only five of which used an independent validation data set. The other studies used the SOFA-based score directly to discriminate between survivors and non-survivors without fitting a probabilistic model. In five of the six studies, admission-based models (Acute Physiology and Chronic Health Evaluation (APACHE) II/III) were reported to have a slightly better discrimination ability than SOFA-based models at admission (the receiver operating characteristic curve (AUC) of SOFA-based models ranged between 0.61 and 0.88), and in one study a SOFA model had higher AUC than the Simplified Acute Physiology Score (SAPS) II model. Four of these studies used the Hosmer-Lemeshow tests for calibration, none of which reported a lack of fit for the SOFA models. Models based on sequential SOFA scores were described in 11 studies including maximum SOFA scores and maximum sum of individual components of the SOFA score (AUC range: 0.69 to 0.92) and delta SOFA (AUC range: 0.51 to 0.83). Studies comparing SOFA with other organ failure scores did not consistently show superiority of one scoring system to another. Four studies combined SOFA-based derivatives with admission severity of illness scores, and they all reported on improved predictions for the combination. Quality of studies ranged from 11.5 to 19.5 points on a 20-point scale.

**Conclusions:**

Models based on SOFA scores at admission had only slightly worse performance than APACHE II/III and were competitive with SAPS II models in predicting mortality in patients in the general medical and/or surgical ICU. Models with sequential SOFA scores seem to have a comparable performance with other organ failure scores. The combination of sequential SOFA derivatives with APACHE II/III and SAPS II models clearly improved prognostic performance of either model alone. Due to the heterogeneity of the studies, it is impossible to draw general conclusions on the optimal mathematical model and optimal derivatives of SOFA scores. Future studies should use a standard evaluation methodology with a standard set of outcome measures covering discrimination, calibration and accuracy.

## Introduction

The development of the Sepsis-related Organ Failure Assessment (SOFA) score was an attempt to objectively and quantitatively describe the degree of organ dysfunction over time and to evaluate morbidity in intensive care unit (ICU) septic patients [1]. Later, when it was realised that it could be applied equally well in non-septic patients, the acronym 'SOFA' was taken to refer to Sequential Organ Failure Assessment [2]. The SOFA scoring scheme daily assigns 1 to 4 points to each of the following six organ systems depending on the level of dysfunction: respiratory, circulatory, renal, haematology, hepatic and central nervous system. Since its introduction, the SOFA score has also been used for predicting mortality, although it was not developed for this purpose.

The aim of this paper was to systematically review, identify research themes and assess studies evaluating the prognostic performance of SOFA-based models (including probabilistic models and simple scores) for predicting mortality in adult patients in medical and/or surgical ICUs.

## Materials and methods

### Search strategy

Two reviewers independently screened the titles and abstracts of articles obtained by the following search procedure. The Scopus database (Jan 1966 to February 2008) was searched for research articles and reviews using the following query: *(critical OR intensive) AND (mortality OR survival) AND (sofa OR "sepsis-related organ failure" OR "sepsis related organ failure" OR "sequential organ failure") *in title, abstract and keywords.

Scopus comprises, among others, clinical databases such as Medline and Embase. Only English language journal articles were considered. In addition, the references of all included articles as well as articles citing them were screened, and authors were approached about follow-up studies in progress. Follow-up studies were only included if they had already been accepted for publication.

### Inclusion criteria

The following inclusion criteria were applied: (1) the study aimed to evaluate a SOFA-based model (probabilistic or as a score); (2) it assessed the statistical performance of the model in terms of accuracy and/or discrimination and/or calibration (studies reporting only on odds ratios and/or standardised mortality ratios were excluded); (3) the predicted outcome of the study was mortality or survival of the patient; and (4) the patient sample was not restricted to a specific diagnosis (e.g. diabetes) but taken from the surgical and/or medical adult ICU population. Two reviewers conducted the search and differences were resolved by consensus after including a third reviewer.

### Quality assessment

The quality of the included studies was assessed based on an adaptation of a quality assessment framework for systematic reviews of prognostic studies [3] [see Additional data file 1]. This framework includes the following six areas of potential study biases: study participation; study attrition; measurement of prognostic factors; measurement of and controlling for confounding variables; measurement of outcomes; and analysis approach. Two reviewers conducted the quality assessment independently from each other and discrepancies were resolved by involving the third reviewer.

### Missing data

Authors were contacted by email to complete missing data that were required for characterising the studies. When the authors did not reply or their answer was still unclear, empty fields were marked with 'Not Reported (NR)'.

### Prognostic performance measures

For each included study we describe the reported discrimination of the model (or score) and if available the reported calibration and accuracy. Discrimination, usually measured in terms of the Area Under the Receiver Operating Characteristic Curve (AUC), refers to a model's ability to assign a higher probability to non-survivors than to survivors. The AUC, however, gives no indication of how close the predicted probabilities are to the true ones (estimated by the observed proportion of death). Calibration refers to this agreement between predicted and true probabilities and is most often measured by the Hosmer-Lemeshow H or C goodness-of-fit statistics (these are based on the chi-squared test). These statistics suggest good fit when the associated p values are greater than 0.05, but they are strongly influenced by sample size. Accuracy is a measure of the average distance (residual) between the observed outcome and its predicted probability for each individual patient. A popular accuracy measure is the Brier score, which is the squared mean of the residual values. The Brier score is sensitive to both discrimination as well as calibration of the predicted probabilities.

## Results

### Search results

**Figure 1:**
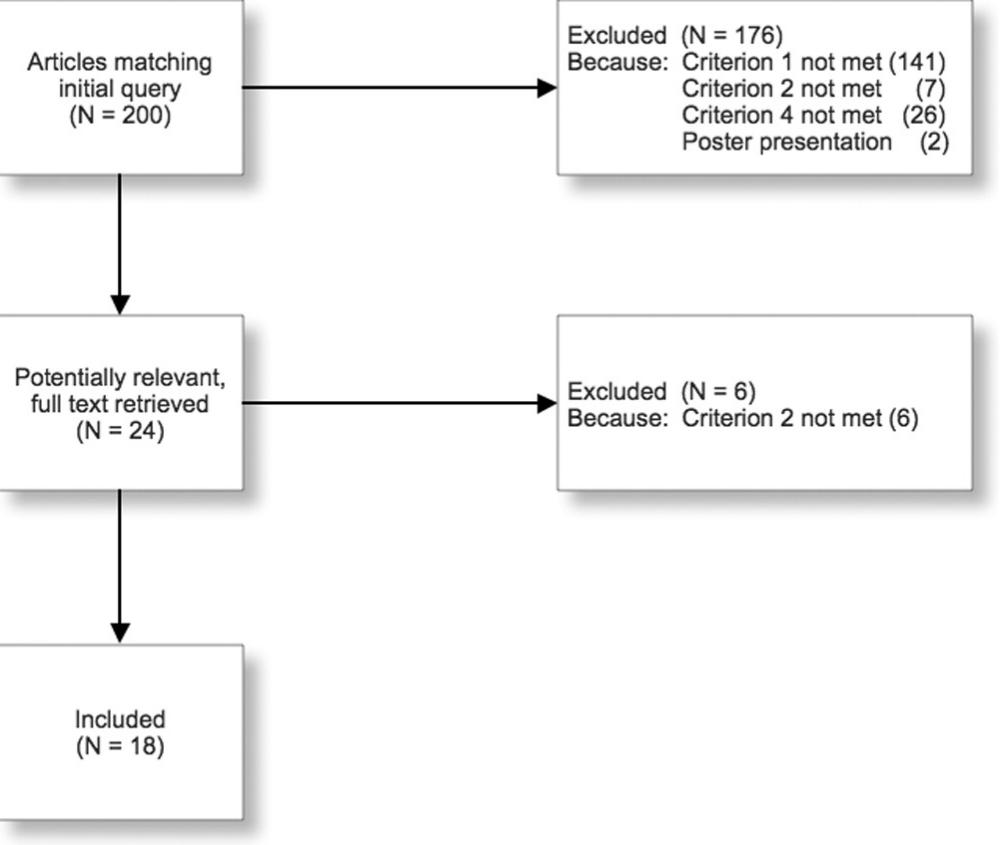
**Search flow chart**. n = Number of studies.

Of 200 studies initially identified, 18 met the inclusion criteria and were included in this study (Figure 1). Inter-observer agreement measured by Kappa was 0.94.

By scanning the reference lists of included articles and those citing them, seven additional articles were rendered potentially relevant. Nevertheless, assessment of their abstracts demonstrated that they did not match our inclusion criteria (six studies did not provide data on discrimination, calibration or accuracy, and one study did not use SOFA to predict mortality).

### Study characteristics

Table 1 shows the characteristics of the included studies. The studies evaluated different types of SOFA derivatives (e.g. mean, maximum) and compared them with different models and covariates. Six studies combined SOFA with other models or covariates [4-9].

**Table 1:** Study characteristics.

	**Study design**		**Population**	**Models**	**Variables**		**Comparison**	
	**Setting (Location)^a^**	**Study period^b^**	**Nc/ICU Typed/Mortality%^e^**	**Model/Valid.^f^**	**SOFA Abstractions^g^**	**Others^h^**	**Standard Model^i^**	**Mort.^j^**

Toma et al (2008) [9]	1 ICU (NL)	Jul 98 to Aug 05	2928/Mix/H = 24	LR/Ind.	Seq of IOF^1^	SAPS II	SAPS II	H
Toma et al (2007) [8]	1 ICU (NL)	Jul 98 to Aug 05	6276/Mix/H = 11	LR/Ind.	Seq of SOFA^2^	SAPS II	SAPS II	H
Ho (2007) [4]	1 multidisc ICU (AU)	Jan 05 to Dec 05	1311/Mix/H = 14.5	LR/Ind.	TMS Adm Delta (TMS-Adm)	APACHE II	APACHE II	H
Ho et al (2007) [11]	1 multidisc ICU (AU)	Jan 05 to Dec 05	1311/Mix/H = 14.5	No	TMS Adm Delta (TMS-Adm)	No	APACHE II, APS, RPH	H
Holtfreter et al (2006) [12]	1 ICU (DE)	42 months	933/Mix/H = 25/I = 23.9	No	Adm	No	16 variables, APACHE II	H
Zygun et al (2005) [14]	3 ICUs (CA)	May 00 to Apr 01	1436/Mix/H = 35.1/I = 27	LR/NR	Adm TMS, Mean (ICU stay), Delta (TMS-Adm), Adm (i)	No	MODS	H/I
Cabré et al (2005) [6]	79 ICUs (75 ES, 4 L-Am)	Feb 01 to Mar 01	1324/Mix/H = 44.6/I = 37.3	LR/NR	Min (MODS period), Max (MODS period), 5-day trend^3^	Age	No	H
Timsit et al (2002) [15]	6 ICUs (FR)	24 months	1685/Mix/H = 30.3/I = 22.5	LR/Ind.*	D1-7, D1-7 (mod)	No	LODS	H
Pettilä et al (2002) [17]	1 med-surg ICU (FI)	NR	520/Mix/H = 30/I = 16.5	No	Adm, D5, Max (5d), Delta (d5-d1), TMS	No	APACHE III, MODS, LODS	H
Janssens et al (2000) [20]	1 med ICU (DE)	Nov 97 to Feb 98	303/Med/H = 14.5/I = 6.3	LR/NR	Adm, TMS, Delta (TMS-Adm)	No	No	H
Khwannimit (2007) [10]	1 ICU (TH)	Jul 04 to Mar 06	1782/Mix/H = 22/I = 16.4	No	Adm	No	MODS, SOFA, LODS	I
Rivera- Fernández et al (2007) [5]	55 ICUs (EU)	2 months in 97/98	6409/Mix/H = 20.6/I = 13.9	LR/Ind.	Mean (ICU stay), Max(ICU stay)	SAPS II, diagnosis events	SAPS II	I
Gosling et al (2006) [13]	1 general ICU (UK)	Nov 02 to Oct 03	431/Mix/I = 20.9	No	Adm SOFA	No	APACHE II, urine albumin and 5 other factors	I
Kajdacsy- Balla Amaral et al (2005) [7]	40 ICUs (1 AU, 35 EU, 1 N-Am, 3 S-Am)	1 May 95 to 31 May 95	748 (6 countries)/Mix/I = 21.5	LR/NR	Adm, TMS, Delta (48 h-Adm), Delta (TMS-Adm)	Different parameters	No	I
Junger et al (2002) [18]	1 operative ICU (DE)	Apr 99 to Mar 00	524/Surg/I = 12.4	No	Max (ICU stay), TMS, Delta (TMS-Adm), Adm (mod)	No	No	I
Ferreira et al (2001) [19]	1 med-surg ICU (BE)	Apr 99 to Jul 99	352/Mix/I = 23	No	Adm, 48 h, 96 h, Delta (48 h-Adm), Delta (96 h-Adm), Max (ICU stay), Mean (ICU stay), Total	No	No	I
Moreno et al (1999) [21]	40 ICUs (1 AU, 35 EU, 1 N-Am, 3 S-Am)	May 95	1449/Mix/H = 26/I = 22	LR/NR	Adm, TMS, Delta (TMS-Adm), Adm (i)	No	No	I
Bota et al (2002) [16]	1 ICU (BE)	Apr to Jul99, Oct to Nov99, Jul to Sep00	949/Mix/U = 29.1	No	Adm, 48 h, 96 h, Dis, Max (24 h), Adm (c), 48 h (c), 96 h (c), Dis (c), Max (c, 24 h)	No	APACHE II, MODS	U

a: AU = Australia, BE = Belgium, CA = Canada, DE = Germany, EU = European Union, ES = Spain, FR = France, FI = Finland, ICU = Intensive Care Unit, L-Am = Latin-America, med = medical, multidisc = multidisciplinary, N-Am = North-America, NL = The Netherlands, S-Am = South-America, surg = surgical, TH = Thailand, UK = United Kingdom.

b: NR = Not reported.

c: N = Number of patients.

d: Med = medical, Mix = Mixed, Surg = surgical.

e: H = Hospital mortality, I = ICU mortality, U = Unspecified mortality.

f: Ind. = Independent validation set used (*indicates the use of bootstrapping), LR = Logistic Regression, Model = Model type reported, No = No model was used, NR = Not Reported, Valid. = Validation method.

g: 1 = Sequences of categorised individual components of SOFA (Failure-Non failure), 2 = Sequences of categorised SOFA scores (High-Medium-Low), 3 = SOFA trend over 5 days (-1 if SOFA is decreased, 0 if SOFA is unchanged, 1 if SOFA is increased), Adm = Admission, c = cardiovascular component of SOFA, cust = customised, Dis = Discharge, Dx = Day x (x = day number), i = individual components of SOFA, IOF = individual Organ Failure scores, Max = Maximum, mod = modified, seq = sequences, SOFA = Sequential Organ Failure Assessment, TMS = Total Maximum SOFA, xd = x days (x = number of days), xh = x hours (x = number of hours).

h: APACHE = Acute Physiology And Chronic Health Evaluation, SAPS = Simplified Acute Physiology Score.

i: APACHE = Acute Physiology And Chronic Health Evaluation, APS = Acute Physiology Score, LODS = Logistic Organ Dysfunction System, MODS = Multiple Organ Dysfunction Score, RPH = Royal Perth Hospital Intensive Care Unit, SAPS = Simplified Acute Physiology Score, SOFA = Sequential Organ Failure Assessment.

j: H = Hospital mortality, I = ICU mortality, Mort. = Mortality, U = Unspecified mortality.

Seventeen studies (94%) measured the AUC [4-7,9-21], four studies (22%) measured the Brier score [4,8,9,11] and six studies (33%) calculated Hosmer-Lemeshow (HL) statistics [4,5,7,11,14,15] (two studies used the C-statistic [4,11], one used the H-statistic [5], one used both [7] and the rest [14,15] did not specify which of the two statistics were used).

Studies were not always clear about the kind of model used to evaluate SOFA. Only 10 studies (56%) reported the use of a logistic regression model [4-9,14,15,20,21]. The models in these studies were fitted on local developmental data sets. Five of these ten studies validated the model on an independent test set [4,5,8,9,15] and five studies did not report how the model was validated [6,7,14,20,21]. Hospital mortality was the outcome in 10 studies [4,6,8,9,11,12,14,15,17,20], ICU mortality in eight studies [5,7,10,13,14,18,19,21] and in one study mortality type was unspecified [16]. One study evaluated both ICU and hospital mortality [14].

#### Missing data

Study characteristics that were most often missing were: type of patient population (surgical/medical/mix); type of model (e.g. logistic regression); and whether the model was validated on the developmental or independent validation set. Emailing the authors confirmed the type of ICU outcome (hospital or ICU mortality) used in one study.

### Study quality

We used four of the six main quality aspects in the framework of Hayden and colleagues [3] leaving 'study attrition' (such as loss to follow-up) and 'confounding measurement and account' out. The former is irrelevant in our analysis and the latter falls outside the scope of this review. The maximum quality score is 20. The results of the quality assessment of the included studies are shown in Table 2.

**Table 2:** Quality score of included studies.

	**Study participation max 8 pts**	**Prognostic factor max 3 pts**	**Outcome measurement max 1 pt**	**Analysis max 8 pts**	**Total score max 20 pts**
Toma et al (2008) [9]	8	3	1	7.5	19
Toma et al (2007) [8]	8	2.5	1	8	19.5
Khwannimit (2007) [10]	8	1	1	3.5	13.5
Ho (2007) [4]	8	3	1	7	19
Ho et al (2007) [11]	8	2	1	5	16
Rivera-Fernández et al (2007) [5]	7	1	1	7.5	16.5
Holtfreter et al (2006) [12]	8	1.5	1	5	15.5
Gosling et al (2006) [13]	8	1.5	1	4	14.5
Zygun et al (2005) [14]	8	2	1	5.5	16.5
Cabré et al (2005) [6]	8	2	1	4	15
Kajdacsy-Balla Amaral et al (2005) [7]	8	3	1	5	17
Timsit et al (2002) [15]	8	2.5	1	7.5	19
Bota et al (2002) [16]	7.5	1	0	3	11.5
Pettilä et al (2002) [17]	8	1	1	7.5	17.5
Junger et al (2002) [18]	7	2	1	3	13
Ferreira et al (2001) [19]	8	2.5	1	3	14.5
Janssens et al (2000) [20]	8	2	1	3.5	14.5
Moreno et al (1999) [21]	8	2.5	1	3.5	15

max = maximum score (criteria for quality assessment are based on a 20 item list [see Additional data file 1]).

### Study results

The cohort size ranged from 303 to 6409 patients. Mean age was 53 to 62 years in complete cohorts and there was a median age of 66 years in one study [15]. The percentage of males was 52% to 71%. Hospital mortality ranged from 11% to 45% and ICU mortality from 6.3% to 37%.

Studies were heterogeneous in the way they used SOFA. The major themes identified in the evaluation studies were investigating the performance of: single SOFA scores at admission or at a fixed time after admission; sequential measurements of SOFA (e.g. mean SOFA score); individual components of SOFA (e.g. cardiovascular component); combination of SOFA with other covariates; and temporal models using patterns discovered in the SOFA scores.

#### Performance of single SOFA scores at a fixed time on and after admission

Eleven studies (61%) evaluated the SOFA score on admission (Table 3) [10-17,19-21]. In seven studies, SOFA on admission was calculated using the most abnormal values from the first 24 hours after admission [10,12,14,16,17,19,20]. Discrimination, measured by the AUC, ranged between 0.61 and 0.88. P values of HL-statistics ranged from 0.17 to 0.8. Four studies (22%) evaluated SOFA on days other than the day of admission [15-17,19]. In these studies, AUCs ranged between 0.727 and 0.897 and p values of HL-statistics ranged between 0.09 and 0.27 for days 2 to 7 after admission and at the day of ICU discharge. Six studies (33%) compared admission SOFA with traditional admission-based models [11-13,16,17,20]. The comparison is more meaningful in the first four studies [11,12,17,20] which, in line with the admission-based models, were developed to predict hospital mortality. Two of those studies reported that the Acute Physiology And Chronic Health Condition (APACHE) II score had better or slightly better discrimination than admission SOFA [11-13]. Furthermore, one study found better calibration for the APACHE II score [11]. This same study also found that the Simplified Acute Physiology Score (SAPS; defined as the APACHE II score without age and chronic health conditions) had comparable discriminative ability to admission SOFA and better calibration. One study reported comparable discrimination (AUC = 0.776 and 0.825 for SOFA and APACHE III, respectively) and comparable calibration for SOFA and APACHE III on admission [17]. Finally, one study reported that admission SOFA had a higher AUC (0.82) than SAPS II (0.77) [20]. In the other two studies that compared admission SOFA with traditional admission-based models, the outcome was either ICU mortality [13] or unspecified [16]. In these two studies the APACHE II score was reported to have slightly better discrimination than, but in essence comparable with, admission SOFA (0.62 versus 0.61 [13] and 0.88 versus 0.872 [16]).

**Table 3:** Performance at admission or a fixed time thereafter.

**Admission SOFA**	**AUC**	**Brier**	**H/C-statistics**	**Compared with**	**AUC**	**Brier**	**H/C-statistics**	**Mort**.
Ho et al (2007) [11]	0.791	0.1	C = 7.97	APACHE II	0.858	0.09		H
			p = 0.437	APS	0.829	0.09	C = 2.9 p = 0.890	H
				RPHICU	0.822	0.09	C = 4.7 p = 0.198	H
Holtfreter et al (2006) [12]	0.72			APACHE II	0.785			H
Zygun et al (2005) [14]	0.67		U = 8.8	MODS	0.62		H/C = 10.28	H
			p = 0.38				p = 0.17	
Timsit et al (2002) [15]	0.72		U = 4.55	LODS	0.726		H/C = 10.4	H
			p = 0.8				p = 0.16	
Pettilä et al (2002) [17]	0.776			APACHE III	0.825			H
				LODS	0.805			H
				MODS	0.695			H
Khwannimit (2007) [10]	0.8786			LODS	0.8802			H
				MODS	0.8606			
Gosling et al (2006) [13]	0.61			APACHE II	0.62			I
Zygun et al (2005) [14]	0.67		U = 11.66	MODS	0.63		H/C = 14.29	I
			p = 0.17				p = 0.05	
Moreno et al (1999) [21]	0.772							I
Bota et al (2002) [16]	0.872			APACHE II	0.88			U
				MODS	0.856			U
Ferreira et al (2001) [19]	0.79							I
Janssens et al (2000) [20]	0.82			SAPS II	0.77			H

**Other scoring moments**	**AUC**	**Brier**	**H/C-statistics**	**Compared to**	**AUC**	**Brier**	**H/C-statistics**	**Mort**.

Bota et al (2002) [16] 48 hours	0.844			MODS	0.834			U
Ferreira et al (2001) [19] 48 hours	0.78							I
Bota et al (2002) [16] 96 hours	0.847			MODS	0.861			U
Ferreira et al (2001) [19] 96 hours	0.82							I
Timsit et al (2002) [15], day 2	0.742		U = 11.1	LODS	0.742			H
			p = 0.2					
Timsit et al (2002) [15], day 3	0.762		U = 9.94	LODS	0.762			H
			p = 0.27					
Timsit et al (2002) [15], day 4	0.766		U = 10.5	LODS	0.766			H
			p = 0.23					
Timsit et al (2002) [15], day 5	0.746		U = 13.6	LODS	0.746			H
			p = 0.09					
Pettilä et al (2002) [17], day 5	0.727			LODS	0.76			H
H					MODS	0.744		
Timsit et al (2002) [15], day 6	0.763		U = 12.2	LODS	0.763			H
			p = 0.14					
Timsit et al (2002) [15], day 7	0.746			LODS	0.764			H
Bota et al (2002) [16], final	0.897			MODS	0.869			H

APACHE = Acute Physiology and Chronic Health Evaluation, APS = Acute Physiology Score (APACHE without chronic health and age condition), AUC = Area Under the Receiver Operating Characteristic Curve, H = Hospital, H/C = H- or C- Hosmer-Lemeshow statistics, I = Intensive care unit, LODS = Logistic Organ Dysfunction System, MODS = Multiple Organ Dysfunction Score, Mort. = Mortality, RPHICU = Royal Perth Hospital Intensive Care Unit, SAPS = Simplified Acute Physiology Score, SOFA = Sequential Organ Failure Assessment, U = Unspecified (mortality type or H/C statistic).

Five studies (28%) compared SOFA with other organ failure scores [10,14-17]. Generally, no clear differences were found in calibration or discrimination (Table 3).

#### Performance of sequential measurements of SOFA

Eleven studies (61%) evaluated sequential measurements of SOFA [7,11,14-21]. The derivatives evaluated were: max SOFA (four studies), total max SOFA (seven studies), delta SOFA (seven studies), mean SOFA (two studies), total SOFA (one study) and modified SOFA (two studies) (Table 4).

**Table 4:** Performance for sequential SOFA.

**Max SOFA**	**AUC**	**Brier**	**H/C-statistics**	**Comp**.	**AUC**	**H/C-statistics**	**Mort**.
Pettilä et al (2002) [17], 5 days	0.792			LODS	0.827		H
				MODS	0.795		H
Junger et al (2002) [18], ICU stay	0.922						I
Bota et al (2002) [16], 24 hrs period	0.898			MODS	0.9		U
Ferreira et al (2001) [19], ICU stay	0.9						I

**Total Max SOFA**	**AUC**	**Brier**	**H/C-statistics**	**Comp**	**AUC**	**H/C-statistics**	**Mort**.

Ho et al (2007) [11], ICU stay	0.829	0.1	C = 7.4 p = 0.496				H
Zygun et al (2005) [14], ICU stay	0.7		U = 9.2 p = 0.33	MODS	0.65	8.07 p = 0.43	H
Pettilä et al (2002) [17], ICU stay	0.816			LODS	0.839		H
				MODS	0.817		H
Zygun et al (2005) [14], ICU stay	0.69		U = 7.30 p = 0.50	MODS	0.64	9.09 p = 0.33	I
Kajdacsy-Balla Amaral et al (2005) [7], ICU stay	0.84		H: p = 0.95 C: p = 0.54				I
Junger et al (2002) [18], ICU stay	0.921						I
Moreno et al (1999) [21], ICU stay	0.847						I
Janssens et al (2000) [20], ICU stay	0.86						H

**Delta SOFA**	**AUC**	**Brier**	**H/C-statistics**	**Comp**	**AUC**	**H/C-statistics**	**Mort**.

Ho et al (2007) [11], TMS – Adm	0.635	0.12	C = 20.2 p = 0.001				H
Zygun et al (2005) [14], TMS – Adm	0.54		U = 53.48 p < 0.01	MODS	0.55	31.2 p < 0.01	H
Pettilä et al (2002) [17], day 5 – Adm	0.6			LODS	0.633		H
				MODS	0.653		H
Zygun et al (2005) [14], TMS – Adm	0.51		U = 98.01 p < 0.01	MODS	0.52	70.52 p < 0.01	I
Junger et al (2002) [18], TMS – Adm	0.828						I
Moreno et al (1999) [21], TMS – Adm	0.742						I
Ferreira et al (2001) [19], 48 hrs – Adm	0.69						I
Ferreira et al (2001) [19], 96 hrs – Adm	0.62						I
Janssens et al (2000) [20], TMS – Adm	0.62						H

**Mean SOFA**	**AUC**	**Brier**	**H/C-statistics**	**Comp**	**AUC**	**H/C-statistics**	**Mort**.

Zygun et al (2005) [14], ICU stay	0.77		U = 22.66 p < 0.01	MODS	0.74	46.13 p < 0.01	H
Zygun et al (2005) [14], ICU stay	0.79		U = 28.92 p < 0.01	MODS	0.75	42.72 p < 0.01	I
Ferreira et al (2001) [19], ICU stay	0.88						I

**Total SOFA**	**AUC**	**Brier**	**H/C-statistics**	**Comp**	**AUC**	**H/C-statistics**	**Mort**.

Ferreira et al (2001) [19], ICU stay	0.85						I

**Modified SOFA**	**AUC**	**Brier**	**H/C-statistics**	**Comp**	**AUC**	**H/C-statistics**	**Mort**.

Timsit et al (2002) [15], Adm	0.729		U = 11 p = 0.2	LODS	0.733	11.3 p = 0.19	H
Timsit et al (2002) [15], day 2	0.752		U = 8.3 p = 0.4	LODS	0.748		H
Timsit et al (2002) [15], day 3	0.773		U = 11.3 p = 0.19	LODS	0.761		H
Timsit et al (2002) [15], day 4	0.779		U = 7.3 p = 0.5	LODS	0.76		H
Timsit et al (2002) [15], day 5	0.763		U = 14.4 p = 0.07	LODS	0.749		H
Timsit et al (2002) [15], day 6	0.784		U = 11 p = 0.17	LODS	0.79		H
Timsit et al (2002) [15], day 7	0.768		U = 6.3 p = 0.62	LODS	0.746		H
Junger et al (2002) [18], Adm	0.799						I

Adm = admission, AUC = Area Under the Receiver Operating Characteristic Curve, Comp. = Compared with, H = Hospital, H/C = H- or C- Hosmer-Lemeshow statistics, hrs = hours, I = ICU = Intensive care unit, LODS = Logistic Organ Dysfunction System, max = maximum, MODS = Multiple Organ Dysfunction Score, Mort. = Mortality, SOFA = Sequential Organ Failure Assessment, TMS = total max SOFA (always measured over entire ICU stay), U = Unspecified (mortality type or H/C statistic).

Total max SOFA was always defined as the sum of the highest scores per individual organ system (e.g. cardiovascular) during the entire ICU stay. Max SOFA always referred to the highest total SOFA score measured in a prespecified time interval, and mean SOFA was always calculated by taking the average of all total SOFA scores in the prespecified time interval. These intervals varied in length, but generally they were equal to the complete ICU stay. Definitions of delta SOFA were not consistent. Generally, delta SOFA was defined as total max minus admission SOFA [4,7,11,14,18,20,21], but some studies used different definitions [7,17,19]. Modified SOFA scores were adapted SOFA scores (e.g. by using a surrogate of the Glasgow Coma Scale).

Best AUCs were found for max SOFA (range = 0.792 to 0.922) and total max SOFA (range = 0.69 to 0.921), and the lowest AUC was found for delta SOFA (range = 0.51 to 0.828). P values of HL-statistics ranged from 0.33 to 0.95 for total max SOFA and were all beneath 0.05, indicating poor fit, for delta SOFA and mean SOFA.

#### Performance of individual components of SOFA

Four studies (22%) evaluated individual components of SOFA [10,14,16,21] (Table 5). The cardiovascular component performed best in one study [21] and the neurological component in another [10], while the hepatic component did worst in both [10,21]. In one study [16], the max cardiovascular component had a higher AUC than the other derivatives of the cardiovascular component.

**Table 5:** Performance for individual components of SOFA.

**Cardiovascular SOFA**	**AUC**	**Compared with**	**AUC**	**Mortality**
Zygun et al (2005) [14], Adm	0.68	MODS	0.63	Hospital
Khwannimit (2007) [10], Adm	0.725	LODS	0.772	ICU
		MODS	0.726	ICU
Zygun et al (2005) [14], Adm	0.74	MODS	0.64	ICU
Moreno et al (1999) [21], Adm	0.802			ICU
Bota et al (2002) [16], Adm	0.75	MODS	0.694	Unspecified
Bota et al (2002) [16], 48 hours	0.732	MODS	0.675	Unspecified
Bota et al (2002) [16], 96 hours	0.739	MODS	0.674	Unspecified
Bota et al (2002) [16], discharge	0.781	MODS	0.75	Unspecified
Bota et al (2002) [16], max	0.821	MODS	0.75	Unspecified

**Respiratory SOFA**	**AUC**	**Compared with**	**AUC**	**Mortality**

Khwannimit (2007) [10], Adm	0.725	LODS	0.704	ICU
		MODS	0.71	ICU
Moreno et al (1999) [21], Adm	0.736			ICU

**Hepatic SOFA**	**AUC**	**Compared with**	**AUC**	**Mortality**

Khwannimit (2007) [10], Adm	0.539	LODS	0.563	ICU
		MODS	0.539	ICU
Moreno et al (1999) [21], Adm	0.655			ICU

**Renal SOFA**	**AUC**	**Compared with**	**AUC**	**Mortality**

Khwannimit (2007) [10], Adm	0.678	LODS	0.727	ICU
		MODS	0.659	ICU
Moreno et al (1999) [21], Adm	0.739			ICU

**Neurological SOFA**	**AUC**	**Compared with**	**AUC**	**Mortality**

Khwannimit (2007) [10], Adm	0.84	LODS	0.822	ICU
		MODS	0.839	ICU
Moreno et al (1999) [21], Adm	0.727			ICU

**Coagulation SOFA**	**AUC**	**Compared with**	**AUC**	**Mortality**

Khwannimit (2007) [10], Adm	0.623	LODS	0.59	ICU
		MODS	0.632	ICU
Moreno et al (1999) [21], Adm	0.684			ICU

Adm = admission, AUC = Area Under the Receiver Operating Characteristic Curve, ICU = Intensive care unit, LODS = Logistic Organ Dysfunction System, max = maximum, MODS = Multiple Organ Dysfunction Score, SOFA = Sequential Organ Failure Assessment.

Studies comparing derivatives of SOFA with similar derivatives of the Logistic Organ Dysfunction System (LODS) score and/or the Multiple Organ Dysfunction Score (MODS) found good, comparable discrimination, showing a similar pattern of performance of the different derivatives [10,14-17]. In one study, however, all derivatives of the cardiovascular component of SOFA did better than that of MODS [16].

#### Performance of SOFA combined with other models and/or covariates

Six studies (33%) evaluated SOFA combined with other models and covariates [[4-7] (Table 6); [8,9] (Table 7)].

**Table 6:** Performance for combined models.

**APACHE II**	**Given by**	**AUC**	**Brier**	**H/C statistics**	**Mortality**
APACHE II	Ho (2007) [4]	0.859	0.09	C = 10 p = 0.189	Hospital

APACHE II + Total Max SOFA	Ho (2007) [4]	0.875	0.086	C = 10.1 p = 0.261	Hospital

APACHE II + Delta SOFA	Ho (2007) [4]	0.874	0.086	C = 7.5 p = 0.485	Hospital

APACHE II + Admission SOFA	Ho (2007) [4]	0.861	0.09	C = 9.3 p = 0.318	Hospital

**SAPS II**	**Given by**	**AUC**	**Brier**	**H/C statistics**	**Mortality**

SAPS II	Rivera-Fernández et al (2007) [5]	0.8			ICU

SAPS II + Diagnosis	Rivera-Fernández et al (2007) [5]	0.84			ICU

SAPS II + Diagnosis + Events	Rivera-Fernández et al (2007) [5]	0.91			ICU

SAPS II + Mean SOFA + Max SOFA + Events	Rivera-Fernández et al (2007) [5]	0.93			ICU

SAPS II + Mean SOFA+ Max SOFA + Events + Diagnosis	Rivera-Fernández et al (2007) [5]	0.95		H: 12.02 p > 0.05	ICU

**Other covariates**	**Given by**	**AUC**	**Brier**	**H/C statistics**	**Mortality**

Min SOFA + Max SOFA+ SOFA trend over 5 days + Age	Cabré et al (2005) [6]	0.807			Hospital

Max SOFA > 13 + Min SOFA > 10 + Positive SOFA trend + Age > 60	Cabré et al (2005) [6]	0.750			Hospital

Max SOFA > 10 + Min SOFA > 10 + Positive SOFA trend + Age > 60	Cabré et al (2005) [6]	0.758			Hospital

Total Max SOFA	Kajdacsy-Balla Amaral et al (2005) [7]	0.841			ICU

Total Max SOFA + Infection	Kajdacsy-Balla Amaral et al (2005) [7]	0.845			ICU

Total Max SOFA + Infection + Age	Kajdacsy-Balla Amaral et al (2005) [7]	0.853		C: p = 0.37	ICU
				H: p = 0.73	

APACHE = Acute Physiology and Chronic Health Evaluation, AUC = Area Under the Receiver Operating Characteristic Curve, ICU = Intensive care unit, max = maximum, min = minimum, SAPS = Simplified Acute Physiology Score, SOFA = Sequential Organ Failure Assessment.

**Table 7:** Performance for temporal models using pattern discovery.

		**Brier score**						
**SAPS II + SOFA**	**Given by**	**Day 1**	**Day 2**	**Day 3**	**Day 4**	**Day 5**	**Day 6**	**Day 7**

Recalibrated SAPS II	Toma et al (2007) [8]	0.059	0.132	0.17	0.18	0.182		

Recalibrated SAPS II	Toma et al (2008) [9]		0.175	0.168	0.198	0.199	0.215	0.23

Temporal SOFA model	Toma et al (2007) [8]	0.058	0.128	0.161	0.171	0.166		

Temporal SOFA model	Toma et al (2008) [9]		0.168	0.17	0.195	0.183	0.206	0.211

Temporal wSOFA model	Toma et al (2008) [9]		0.166	0.175	0.199	0.19	0.21	0.224

Temporal IOF model	Toma et al (2008) [9]		0.161	0.166	0.187	0.175	0.195	0.216

		**AUC**						

**SAPS II + SOFA**	**Given by**	**Day 1**	**Day 2**	**Day 3**	**Day 4**	**Day 5**	**Day 6**	**Day 7**

Recalibrated SAPS II	Toma et al (2008) [9]		0.761	0.746	0.692	0.66	0.643	0.645

Temporal SOFA model	Toma et al (2008) [9]		0.786	0.780	0.713	0.737	0.690	0.722

Temporal wSOFA model	Toma et al (2008) [9]		0.794	0.771	0.699	0.709	0.672	0.664

Temporal IOF model	Toma et al (2008) [9]		0.794	0.785	0.727	0.740	0.738	0.715

AUC = Area Under the Receiver Operating Characteristic Curve, IOF = Individual Organ Failure, SAPS = Simplified Acute Physiology Score, SOFA = Sequential Organ Failure Assessment, wSOFA = weighted SOFA score.

One study compared the APACHE II model alone to APACHE II combined with each one of total max SOFA, delta SOFA and admission SOFA [4]. Overall performance and discrimination were both improved by the addition of total max SOFA and of the delta SOFA, especially in emergency ICU admissions. Three studies compared the SAPS II model to the SAPS II model when combined with additional information [5,8,9]. One study found that the discriminative ability of SAPS II could be improved by combining it with mean and max SOFA scores, event information and diagnosis information [5]. Two studies built temporal SOFA models and are described in the next section [8,9].

Two studies combined SOFA with other covariates [6,7]. The first study evaluated different combinations of SOFA derivatives and age [6]. Highest discriminative ability (AUC = 0.807) was found with the combination of age, min SOFA, max SOFA and SOFA trend (using the categories increased, unchanged and decreased) over five days. The second study compared a model based on max SOFA alone with a model including max SOFA and infection, and a model including max SOFA, infection and age [7]. The last model had very good calibration and discrimination, and outperformed the model based on max SOFA alone.

#### Performance of temporal SOFA models using pattern discovery

Two studies (11%) by the same research group used pattern discovery to develop temporal models including SAPS II and SOFA data [8,9] (Table 7). The first study used a data-driven algorithm to discover frequent sequences of SOFA scores, categorised as low, medium and high [8]. On all days examined (the first five days) the temporal SAPS II model including the frequent SOFA patterns (called episodes) had better accuracy, indicated by lower Brier scores, than the original model. On days 2, 4 and 5 these differences were statistically significant. In the second study the same algorithm was used to discover frequent patterns of individual organ failure (IOF) scores (categorised as failure or non-failure) [9] for days 2 to 7. A temporal SAPS II model including the frequent IOF patterns was compared with the original (recalibrated) model, the temporal SAPS II model [8] and a temporal SAPS II model including a weighted average of the SOFA scores. Except for day 7 the model including frequent IOF patterns performed best in terms of both discrimination and accuracy as measured by the AUC and the Brier score [9].

## Discussion

To our knowledge this is the first systematic review on the use of SOFA-based models to predict the risk of mortality in ICU patients. In this review, we show that although the 18 identified studies all focused on evaluating a SOFA-based score or model in predicting mortality they widely differed in the SOFA derivatives used, the time after admission on which the prediction was made, the outcome (hospital or ICU mortality), the prognostic performance measures considered, the way a study was reported and the way the models were validated. This hampers the quantitative comparability of study results. Despite the fact that most studies scored well on most methodological quality dimensions, model validation still formed a weak spot: in some studies there was no report on how performance measures were obtained and in others there was no independent validation set used. The AUC of SOFA-based models was good to very good and did not lag much behind APACHE II/III and was competitive with a SAPS II model. When reported, the Hosmer-Lemeshow tests did not indicate poor fit (i.e. there were no significant departures between the predicted probabilities and the respective observed mortality proportions). Models with sequential SOFA seem to have comparable performance with other organ failure scores. Combining SOFA-based derivatives with admission severity of illness scores clearly improved predictions.

Among the used SOFA derivatives are the SOFA score on admission, maximum SOFA score over the entire ICU stay or the sum of highest SOFA components over ICU stay. Only 10 studies reported on the use of SOFA derivatives as covariates in a logistic regression model, the other eight studies did not use models or did not report on such use. The score itself, without using a probabilistic model would allow for obtaining an AUC representing the likelihood that a non-surviving patient would have a higher SOFA score than a patient that would survive. As the SOFA score itself does not give a quantitative estimation of the risk of mortality, calibration and accuracy cannot be assessed for the SOFA score itself. Remarkably, only 5 of the 10 studies fitting a logistic regression model reported on the use of an independent data set to validate the model. Due to these differences in the use of SOFA scores and in the methodological approach and quality, results of individual studies are very difficult to compare and meta-analyse.

Most studies evaluated prognosis based on SOFA scores in the first 24 hours after ICU admission. Good to excellent discrimination between survivors and non-survivors were reported, which did not markedly differ from that of traditional models such as APACHE II or SAPS II. This relatively good performance of SOFA is remarkable, given the fact that SOFA is based on fewer physiological parameters and that it does not include information on reason for admission or co-morbidity. On the other hand, information on instituted treatments, such as vasopressors and mechanical ventilation, is included in SOFA but not in APACHE II or SAPS II. We would like to stress that SAPS and APACHE models were developed for predicting hospital mortality, hence when comparing SOFA-based models to this family of admission-based models it is more appropriate to use hospital mortality rather than ICU mortality as the outcome. Table 1 shows that this design principle was not always followed.

It can be expected that adding information on the course of the ICU treatment, as reflected by sequential SOFA scores, will improve the accuracy of predicting the likelihood of survival. Indeed, studies that evaluated the prognostic value of highest SOFA scores during ICU stay found excellent discrimination as reflected in high AUCs. It should be stressed, however, that most severe IOF and highest SOFA scores might well be found just before death. The clinical relevance of predicting a high likelihood of dying just before actual death is limited. Interestingly, the one study that evaluated max SOFA over the first five days of admission instead of over the entire ICU stay found an AUC of 0.79, which was almost the same as the AUC for a single SOFA-score at admission [17].

A high delta SOFA indicates increasing organ dysfunction during ICU stay, and was expected to be highly predictive of mortality. In contrast, discrimination of survivors from non-survivors by delta SOFA alone appeared to be poor. This may be explained by the fact that delta SOFA may be relatively low in patients with an already very high SOFA score at admission. Furthermore, delta SOFA does not take into account whether organ functioning improves after the SOFA score reaches a peak value.

Combining information of severity of illness at admission and information on the course of illness during treatment, in contrast to comparing them, seems promising and two strategies have been adopted. In the first strategy a prognostic model at admission was combined with a pre-specified SOFA derivative such as delta SOFA or max SOFA. Indeed, in our review we found that the studies combining delta SOFA or max SOFA with APACHE II or SAPS II reported on better discrimination between survivors and non-survivors for the combined models than for either APACHE II or SAPS II alone [4,5]. A second strategy is to combine severity of admission scores with data-driven patterns of SOFA or individual organ failure scores (e.g. two days of renal failure accompanied with recovery of the neurological system) instead of using pre-specified SOFA derivatives. Two studies adopted this strategy and showed that models based on SAPS II and temporal patterns outperformed models using the SAPS II score alone but recalibrated per day [8,9].

## Conclusion

Interest in models based on the SOFA score, introduced a decade ago, is increasing in recent years. Although the heterogeneity of published studies hampers drawing precise conclusions about the optimal derivatives of SOFA scores, the following general conclusions may be drawn. Models based on SOFA scores at admission seem to be competitive with severity of illness models limited to the first 24 hours of admission. Performance of models based on sequential SOFA scores is comparable with that of other organ failure scores. Based on current evidence we advocate the combination of a traditional model based on data from the first 24 hours after ICU admission (e.g. APACHE IV) with sequential SOFA scores (e.g. max SOFA or a SOFA score pattern over a specified time interval). Such a model should be validated in a large independent dataset.

## Key messages

• SOFA-based models evaluated on their prognostic performance fell under the categories: single SOFA scores at fixed times; sequential SOFA measurements; individual SOFA components; combination of SOFA with other covariates; and SOFA patterns automatically discovered from the data.

• For predicting mortality SOFA-based models at admission seem to be competitive with severity of illness models limited to the first 24 hours of admission, and models based on sequential SOFA scores have comparable performance with other IOF scores.

• The combination of SOFA-based models with admission-based models results in superior prognostic performance than each model alone.

• Studies should use an independent validation set to assess performance and should apply multiple performance measures preferably covering discrimination, calibration and accuracy.

## Abbreviations

APACHE: Acute Physiology And Chronic Health Condition; AUC: Area Under the Receiver Operating Characteristic Curve; HL statistics: Hosmer-Lemeshow statistics; ICU: intensive care unit; IOF: individual organ failure; LODS: Logistic Organ Dysfunction System; MODS: Multiple Organ Dysfunction Score; SAPS: Simplified Acute Physiology Score; SOFA: Sequential Organ Failure Assessment.

## Competing interests

The authors declare that they have no competing interests.

## Authors' contributions

LM carried out the search queries, reviewed the articles, assessed their quality and drafted the paper. AAH conceived of the study, reviewed the articles and participated in its design and coordination and helped to draft the manuscript. EdJ assessed the quality of the studies and participated in its design and coordination and helped to draft the manuscript. All authors read and approved the final manuscript.

## Supplementary Material

Additional file 1 a PDF file containing a list that describes the 20 items of the quality assessment framework. Click here for file
