# Supplementary material for: Evaluation of SOFA-based models for predicting mortality in the ICU: A systematic review
Source: Crit Care. 2008 Dec 17;12(6):R161. doi: 10.1186/cc7160 (PMC2646326; doi:10.1186/cc7160)
Supplement: Additional file 1 — a PDF file containing a list that describes the 20 items of the quality assessment framework. [file cc7160-S1.pdf]

Appendix 1 - Quality Assessment Framework

1. Study participation
   (a) Study population described
      i. Description of setting & study period yes/partly/no
      ii. Description of in- and exclusion criteria yes/partly/no
      iii. Description of patient mix (surgical/medical/both) yes/partly/no
      iv. Number of patients reported yes/partly/no
      v. Number of patients > 100 yes/no
      vi. Mortality rate reported yes/partly/no
      vii. Description of patient characteristics (e.g. mean/median age, apache, saps scores etc.) yes/partly/no
   (b) Study population represents source population yes/partly/no

2. Prognostic factor measurement
   (a) Prognostic factors defined
      i. Clear definition of all prognostic factor(s) evaluated (SOFA, SOFA derivatives, moment of measurement, other covariates) yes/partly/no
      ii. Clear description of type of model(s) (e.g. logistic regression) yes/partly/no
   (b) Prognostic factors measured appropriately
      i. Description of proportion of participants with complete data and handling of missing values yes/partly/no

3. Outcome measurement
   (a) Outcome defined
      i. Clear definition of outcome of interest (e.g. ICU/hospital mortality) yes/partly/no

4. Analysis
   (a) Analysis described & appropriate
      i. Description of all evaluation measures yes/partly/no
      ii. Description of model building strategy (e.g. logistic regression) yes/partly/no
      iii. Description of test method (e.g. train/test set, bootstrapping) yes/partly/no

iv. Both aspects of discrimination and calibration evaluated yes/no
v. Separate test set used for testing yes/no

(b) Analysis provides sufficient presentation of data
   i. There is sufficient presentation of data to assess the adequacy of the analysis (e.g. model coefficients with p-values) yes/partly/no
   ii. There is no selective reporting of results yes/partly/no
   iii. There is comparison to or combination with a standard model (admission-based or other organ failure score) yes/partly/no
